# Comparisons of the effects of two types of titratable mandibular advancement devices on respiratory parameters and upper airway dimensions in patients with obstructive sleep apnea: a randomized controlled trial

**DOI:** 10.1007/s00784-023-04945-z

**Published:** 2023-03-17

**Authors:** Xiaoxin Shi, Frank Lobbezoo, Hui Chen, Boudewijn R. A. M. Rosenmöller, Erwin Berkhout, Jan de Lange, Ghizlane Aarab

**Affiliations:** 1 Department of Orofacial Pain and Dysfunction, Academic Centre for Dentistry Amsterdam (ACTA), University of Amsterdam and Vrije Universiteit Amsterdam, Amsterdam, 1081 LA the Netherlands; 2 Department of Oral Radiology, Academic Centre for Dentistry Amsterdam (ACTA), University of Amsterdam and Vrije Universiteit Amsterdam, Amsterdam, 1081 LA the Netherlands; 3 Department of Oral and Maxillofacial Surgery, Academic Centre for Dentistry Amsterdam (ACTA) and Amsterdam University Medical Centers, Amsterdam, the Netherlands; 4 Department of Orthodontics, School and Hospital of Stomatology, Cheeloo College of Medicine, Shandong University & Shandong Key Laboratory of Oral Tissue Regeneration & Shandong Engineering Laboratory for Dental Materials and Oral Tissue Regeneration, No. 44-1 Wenhua Road West, Jinan, 250012 Shandong China

**Keywords:** Obstructive sleep apnea, Mandibular advancement device, Freedom of mandibular vertical opening, Cone beam computed tomography, Upper airway dimensions

## Abstract

**Objectives:**

To compare the effects of two types of titratable mandibular advancement devices (MADs), namely MAD-H (allowing limited vertical opening) and MAD-S (allowing free vertical opening), on respiratory parameters and upper airway dimensions in patients with mild to moderate obstructive sleep apnea (OSA).

**Materials and methods:**

Patients with mild to moderate OSA (5 ≤ apnea–hypopnea index (AHI) < 30 /h) were randomly assigned to two parallel MAD groups. All MADs were subjectively titrated according to a standardized protocol during a 3-month follow-up. Every patient underwent two polysomnographic recordings, and two cone beam computed tomography scans in supine position: one at baseline and another one after 3 months with the MAD in situ. The primary outcome variables were the AHI in supine position (AHI-supine) and the minimal cross-sectional area of the upper airway in supine position (CSAmin-supine).

**Results:**

A total of 49 patients were recruited, and 31 patients (21 men and 10 women) with a mean (± SD) age of 48.5 (± 13.9) years and a mean AHI of 16.6 (± 6.7) /h completed the study. In the per-protocol analysis, there was no significant difference between MAD-H (*n* = 16) and MAD-S (*n* = 15) in their effects on AHI-supine (*P* = 0.14) and CSAmin-supine (*P* = 0.59). Similar results were found in the intention-to-treat analysis (*P* = 0.47 and 0.57, respectively).

**Conclusions:**

Within the limitations of this study, we conclude that there is no significant difference in the effects of an MAD allowing limited vertical opening and an MAD allowing free vertical opening on respiratory parameters and upper airway dimensions in patients with mild to moderate OSA.

**Clinical relevance:**

MADs allowing limited vertical opening and allowing free vertical opening have similar effects on respiratory parameters and upper airway dimensions in patients with mild to moderate OSA.

Trial registration: ClinicalTrials.gov Identifier: NCT02724865. https://clinicaltrials.gov/ct2/show/NCT02724865

## Introduction

Obstructive sleep apnea (OSA) is characterized by recurrent obstructions of the upper airway, often resulting in oxygen desaturations and arousals from sleep [1]. Excessive daytime sleepiness and fatigue, lack of concentration, and loud snoring reported by the patient’s bed partner are frequently reported complaints [2, 3]. Furthermore, patients with untreated OSA are at increased risk of, among others, hypertension, stroke, heart failure, diabetes, and involvement in car accidents [4–7]. The diagnosis of OSA relies on the combination of symptoms, clinical signs, and objective assessment of obstructive respiratory events. The gold standard for assessing respiration during sleep in patients with OSA is full-night polysomnography (PSG). One commonly used indicator of OSA severity is the number of respiratory events (apneas and/or hypopneas) per hour of sleep during PSG recording, viz., apnea–hypopnea index (AHI). An adult showing an AHI of at least 5 events/hour is diagnosed with OSA [1]. Based on the AHI, OSA severity for adults is classified as mild (5 ≤ AHI < 15 events/hour), moderate (15 ≤ AHI ≤ 30 events/hour), or severe (AHI > 30 events/hour) [1].

Continuous positive airway pressure (CPAP) is the most efficacious therapy for OSA [8]. However, the equipment is relatively cumbersome, which results in low compliance of patients [9]. Alternatively, mandibular advancement device (MAD) therapy is recommended as a primary treatment option in patients with mild and moderate OSA, and in patients with severe OSA who refuse or are unable to tolerate CPAP therapy [10–12].

There is a large variety of commercially available customized MADs. Over the years, the customized MAD has evolved from the “mono-bloc” type of appliance, which consists of a single piece giving the mandible a fixed position, towards the current “bi-block titratable” type, which consists of two separate pieces that are dynamically interconnected allowing different degree of lateral and/or vertical movements. Understanding the treatment efficacy and working mechanism of MADs with different design features has an ongoing interest in the clinic [13, 14]. Previous studies [15–19] suggested that mono-block MADs may have higher effectiveness than bi-block titratable MADs because the mandible is protruded firmly. However, a bi-block titratable MAD is more comfortable for patients as it allows some degree of mandibular movement and has higher compliance [19]. Besides, it is easier and takes less time to titrate a bi-block titratable MAD to the optimal mandibular position. Therefore, bi-block titratable MADs are increasingly recommended [20].

Bi-block titratable MADs may differ in the freedom of mandibular vertical opening. There are two types of bi-block titratable MADs that are commonly used in the treatment of OSA: the Herbst appliance (MAD-H) and the SomnoDent appliance (MAD-S). They predominantly differ in the freedom of vertical opening: the MAD-H only allows limited vertical opening, while the MAD-S allows free vertical opening of the mandible during sleep. A retrospective study suggested no significant difference between MAD-H and MAD-S in changing the AHI [21]. However, that study was not randomized, and therefore the outcomes may be biased. As most previous studies focused on the comparisons between mono-bock MADs and bi-block MADs, comparisons of bi-block MADs with different freedom of mandibular vertical opening are limited. Therefore, more evidence is needed on the comparison between these two types of MAD.

The working mechanism of MAD is related to the improvement of the upper airway dimensions through mandibular protrusion [22–24]. On the other hand, mouth opening is unavoidable with MAD in situ, which may decrease the beneficial effects on the volume and the cross-sectional area (CSA) of the upper airway [25–27]. A systematic review study [28] has suggested that a small minimal cross-sectional area of the upper airway (CSAmin) is the most relevant anatomical characteristic of the upper airway related to the pathogenesis of OSA. However, when comparing different MADs, few studies have provided insights into their effects on the upper airway dimensions using three-dimensional images. The investigations on upper airway dimensions may allow better understanding of the working mechanism of MAD and help to explain the different treatment efficacy between MADs.

We hypothesized that MAD-H (allowing limited vertical opening) would lead to better improvement in respiratory parameters and upper airway dimensions compared to MAD-S (allowing free vertical opening), especially in supine position. This hypothesis was based primarily on the notion that gravity may promote more mandibular opening in the MAD-S group in the supine position, which subsequently results in less improvement of the upper airway CSAmin [25, 26] and AHI-supine [29, 30] compared with the MAD-H group. Therefore, the aim of this randomized controlled trial (RCT) was to compare the effects of MAD-H and MAD-S on respiratory parameters and upper airway dimensions in patients with mild to moderate OSA.

## Material and methods

### Overview

This study was a multi-center RCT with a parallel design, in which the effects of two types of titratable MADs on respiratory parameters and upper airway dimensions were compared. The allocation sequence was automatically generated through an electronic data capture system (Castor EDC), using random block size of either 4, 6, or 8 with an allocation ratio of 1:1. The Medical Research Ethics Committee of the Academic Medical Center Amsterdam (AMC) approved this study (#: NL44085.018.13). The study was registered at clinicaltrials.gov (ClinicalTrials.gov identifier: NCT02724865). Written informed consent was obtained from all participants.

### Participants

Eligible patients, diagnosed with OSA at four sleep centers in the Netherlands (Onze Lieve Vrouwe Gasthuis Ziekenhuis (OLVG), Nederlands Slaap Instituut, Medisch Centrum Jan van Goyen, and AMC), were referred to the department of Oral and Maxillofacial Surgery of AMC to participate in the present study. The inclusion criteria were: 1. ≥ 18 years old; 2. ability to speak, read, and write Dutch; 3. ability to follow-up; 4. ability to use a computer with internet connection for online questionnaires; 5. diagnosis with symptomatic mild or moderate OSA (5 ≤ apnea–hypopnea index (AHI) ˂ 30 events/hour) with at least two OSA symptoms (e.g., snoring, fragmented sleep, witnessed apneas, and/or excessive daytime sleepiness determined by Epworth Sleepiness Scale (ESS) [1]); and 6. expected to maintain current lifestyle (e.g., sports, medicine, diet, etc.). The exclusion criteria were: 1. untreated periodontal problems, dental pain, and/or a lack of retention possibilities for an MAD; 2. medication usage that could influence respiration or sleep; 3. evidence of respiratory/sleep disorders other than OSA ( e.g., central sleep apnea syndrome); 4. systematic disorders based on medical history and examination (e.g., rheumatoid arthritis); 5. severe temporomandibular disorders based on a functional examination of the masticatory system; 6. coexistence of non-respiratory sleep disorders (e.g., insomnia, periodic limb movement disorder, or narcolepsy); 7. known medical history of mental retardation, memory disorders, or psychiatric disorders; 8. reversible morphological upper airway abnormalities (e.g., enlarged tonsils, deviated nasal septum, and/or inferior nasal turbinate hypertrophy); 9. inability to provide informed consent; 10. simultaneous use of other modalities to treat OSA; and/or 11. previous treatment with an MAD.

### MADs

**Fig. 1:**
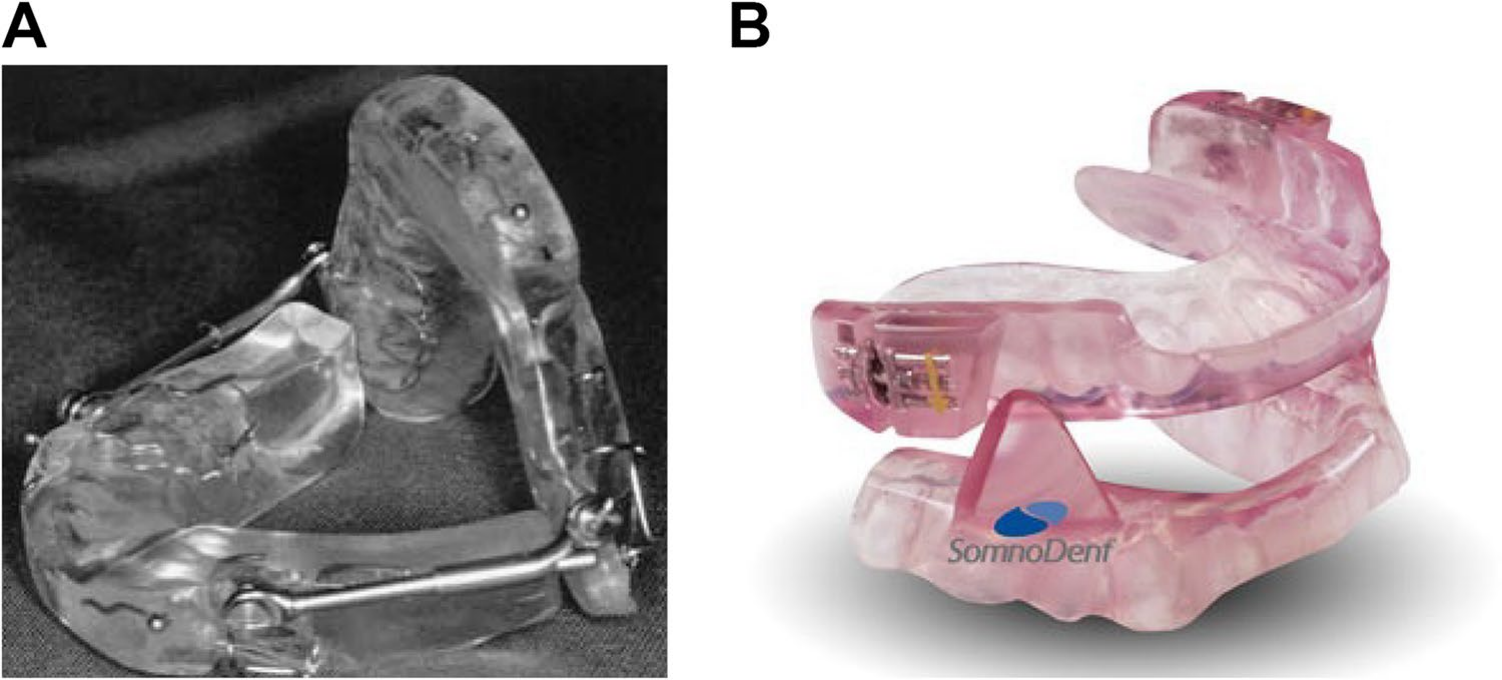
Mandibular advancement devices (MADs). (**A**) MAD-H (Herbst appliance; 4Dental labs, Amsterdam, the Netherlands). (**B**) MAD-S (SomnoDent appliance; SomnoDent Flex, SomnoMed, Sydney, Australia)

The two types of MAD compared in this study were MAD-H (Herbst appliance; 4Dental labs, Amsterdam, the Netherlands) and MAD-S (SomnoDent appliance; SomnoDent Flex, SomnoMed, Sydney, Australia). The MAD-H consisted of two splints connected to each other with adjustable iron-bars for titration. The vertical opening was limited by these bars (Fig. 1A). The MAD-S consisted of two separate splints allowing free vertical opening. It had a screw mechanism on the upper splint which was used for titration (Fig. 1B).

The standardized titration [31] of the MAD was performed at the department of Orofacial Pain and Dysfunction of the Academic Centre for Dentistry Amsterdam (ACTA) for the MAD-S, and the department of Oral and Maxillofacial Surgery of AMC for the MAD-H. All MAD providers were trained for the standardized titration protocol before the start of the study. After adequate assessment of the central relation and maximum protrusion using the George Gauge system with a standard 5-mm vertical dimension (Great Lakes Orthodontics, Tonawanda, NY), MADs were set at 60% of the maximal mandibular protrusion at baseline [31]. The patient returned to the clinic at 4, 8, and 12 weeks after placement of the MAD for titration. At each consecutive visit, the MADs were evaluated and advanced to 75% and 90% if subjective improvement (e.g., perceived reduction of snoring or excessive daytime sleepiness) of OSA was not reached. On the other hand, if side effects were not acceptable for the patient (e.g., tooth pain or signs of temporomandibular disorders), the advancement was adjusted backwards in steps of 15% per visit to 75%, 60%, or 45%, depending on the actual position at the time of the clinical visit. No adjustments were made when the patient reported a sufficient efficacy without side effects.

### MAD side-effects and compliance

The self-reported side effects were recorded at each clinical visit, including the following: 1. sensitive teeth in the morning; 2. painful jaw muscles; 3. painful temporomandibular complaints; and 4. changed occlusion in the morning. The compliance information was collected by a telephone survey, which consisted of four questions: 1. number of hours of MAD use per night; 2. number of hours of total sleep per night; 3. number of days of MAD use per week; and 4. the overall level of satisfaction of the MAD usage. Level of satisfaction was based on a visual analogue scale of 0 to 100, where 0 was unsatisfied, and 100 was very satisfied. The compliance data were expressed as percentage of hours of MAD use per total sleep time, and as percentage of days of MAD use per week.

### Polysomnography (PSG) recordings

Every patient underwent a baseline PSG recording and a 3-month follow-up PSG recording with MAD in situ at one of the afore-mentioned sleep centers. A digital PSG system (Embla A_10_, Broomfield, CO, USA) was used and recorded electroencephalogram (EEG) (FP2-C4/C4-O2), electrooculogram (EOG), electrocardiogram (ECG), and submental and anterior tibial electromyogram (EMG). Nasal airflow was measured by a nasal pressure cannula, and blood oxygen saturation was measured by finger pulse oximetry. Straps containing piezoelectric transducers recorded thoracoabdominal motion, and a position sensor (Sleepsense, St Charles, IL, USA) attached to the midline of the abdominal wall was used to differentiate between supine, prone, right lateral, left lateral, and upright positions [32, 33].

Sleep and respiration were analyzed following the criteria of the American Academy of Sleep Medicine (AASM) Task Force [34]. All polysomnographic variables were scored manually by scorers blinded to the MAD type. Sleep outcome variables included total sleep time (TST), time spent stage N1, stage N2, stage N3, and stage rapid-eye-movement (REM), time spent in supine position, and arousal index. An apnea was defined as the cessation of nasal airflow of more than 90% for a period of ≥ 10 s in the presence of respiratory efforts. A hypopnea was scored whenever there was a > 30% reduced oronasal airflow for at least 10 s, accompanied by ≥ 3% oxygen desaturation from pre-event baseline or an arousal. Respiratory outcome variables included AHI, AHI-supine, AHI-non-supine, and oxygen desaturation index (ODI). Responders were defined as ≥ 50% reduction in baseline AHI with a residual AHI < 10 events/h at the time of therapy evaluation; otherwise, patients were regarded as non-responders [35]. In this study, the AHI-supine was considered as the primary outcome variable for the respiration.

### Cone beam computed tomography (CBCT)

All patients underwent two CBCT scans (NewTom 5G, QR systems, Italy) at the department of Oral Radiology of ACTA: a baseline scan and a follow-up scan with the MAD in situ (in the same protrusion position as during the follow-up PSG recording). The exposure settings were 110 kV, 4 mA, 0.3 mm voxel size, 3.6-s exposure time (pulsed radiation), and 18–36-s scanning time, depending on the size of the patient [36]. At baseline, patients were instructed to maintain light contact between the molars in natural occlusion, while at the follow-up scan, patients were instructed to relax their masticatory muscles with the MAD in situ. CBCT scans were performed in supine position while patients were awake. The head of the patient was positioned with the Frankfort horizontal plane (a plane joining the anatomical landmarks porion (Po) and orbitale (Or)) being perpendicular to floor. After scanning, further standardization of head position was performed, during which the palatal plane (anterior nasal spine (ANS)-posterior nasal spine (PNS)) was adjusted to be parallel to the horizontal plane in the sagittal view and perpendicular to the horizontal plane in the axial view [36].

**Fig. 2:**
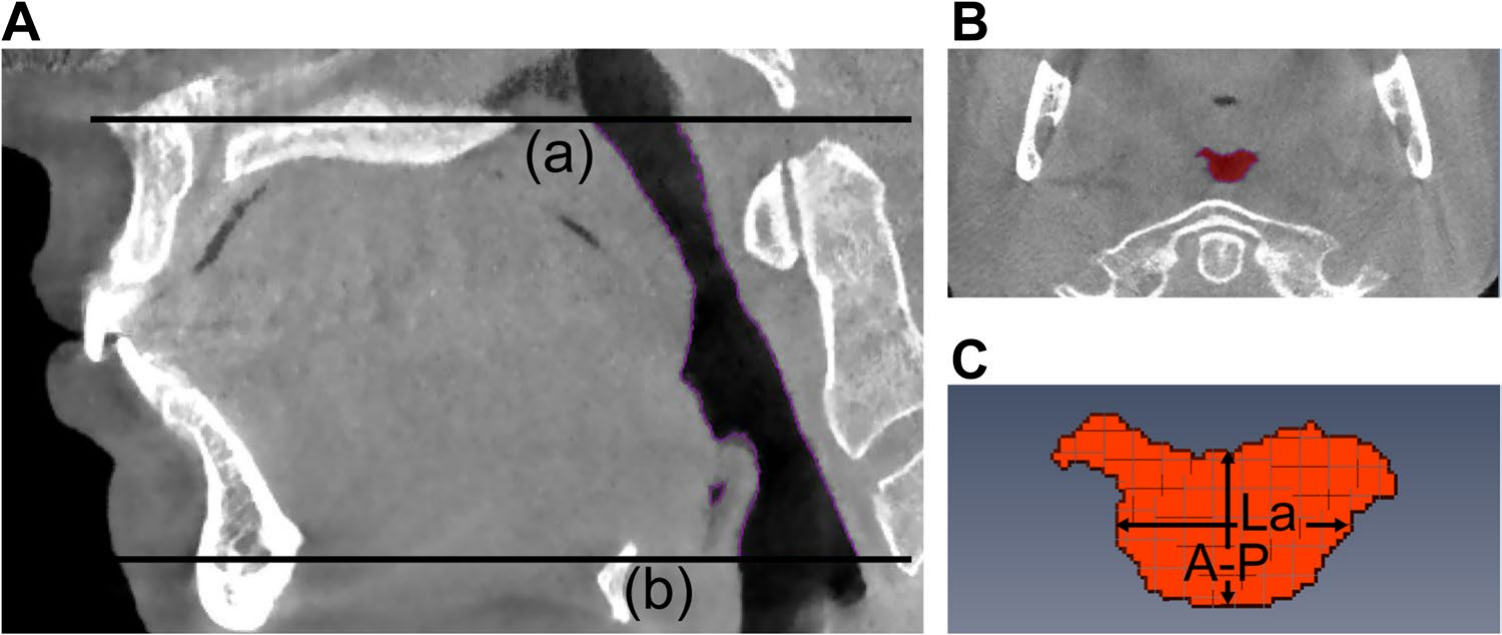
Measurements of the upper airway dimensions using cone beam computed tomography (CBCT) imaging. (**A**) The boundaries of the upper airway from the hard palate (a) to the base of the epiglottis (b) in the sagittal plane. (**B**) The location of the minimal cross-sectional area of the upper airway (CSAmin) in the axial plane. (**C**) The measurements of the anterior–posterior dimension (A-P) and lateral dimension (La) of the CSAmin

Using Amira® software (v4.1, Visage Imaging Inc., Carlsbad, CA, USA), upper airway segmentation was performed by applying the superior and inferior boundaries of the upper airway. The superior boundary of the upper airway was the palatal plane, and the inferior boundary was the horizontal plane across the base of the epiglottis (parallel to the palatal plane) (Fig. 2A). After upper airway segmentation, the upper airway volume (V) and the cross-sectional area (CSA) of each slice were calculated automatically in the software. Based on the results of CSA, the minimum CSA(CSAmin) could be identified and located (Fig. 2B). On the specific slice where the CSAmin was located, the anterior–posterior dimension (A-P) and lateral dimension (La) of CSAmin were measured by the observer, using the linear measuring tool integrated in the software (Fig. 2C). The length of upper airway (L) was calculated by multiplying the slice numbers of the upper airway with 0.3 mm (the thickness of every slice) [36]. As a small CSAmin is the most relevant anatomical characteristic of the upper airway related to the pathogenesis of OSA [28], the CSAmin was selected as the primary outcome variable of upper airway dimensions.

All of the upper airway variables were measured by an experienced examiner. To test the intra-rater reliability of the assessment of the upper airway, 10 CBCT scans were randomly selected and re-measured after a 1-month interval of the original measurements.

**Fig. 3:**
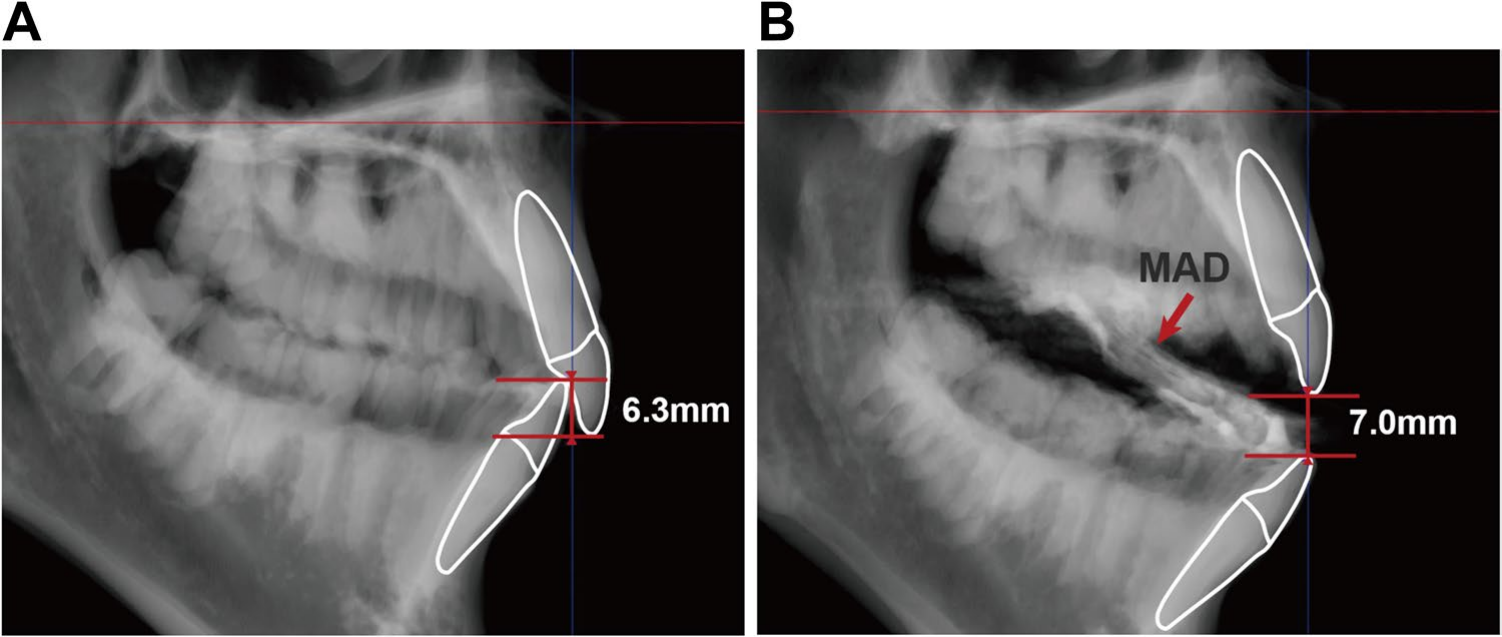
Measurements of mandibular vertical opening using cone beam computed tomography (CBCT) imaging. (**A**) Measurement of overbite (6.3 mm in this example). (**B**) Measurement of the vertical distance between the tip of upper and lower incisors with MAD in situ (7.0 mm in this example). In this case, the vertical opening with MAD in situ is 13.3 mm (i.e., sum of the 6.3 mm and 7.0 mm)

Based on baseline and follow-up CBCT images, the vertical opening with the MAD in situ was determined in two steps in 3Diagnosys ® software (v5.3.1, 3diemme, Cantu, Italy). Firstly, the overbite was measured based on baseline CBCT images (Fig. 3A). Secondly, the vertical distance between the tip of upper and lower incisor with the MAD in situ was measured based on follow-up CBCT images (Fig. 3B). All vertical distances were measured as the perpendicular distance to palatal plane. By adding these two values, the mandibular vertical opening with MAD in situ was determined.

### Sample size

According to the guidelines of Cohen, an effect size d, which is defined as a standardized difference in the means of an outcome variable between two groups, of 0.20 is small, one of 0.5 is medium, and one of 0.8 is large [37]. In our study, we assumed a large effect size (i.e., a large difference between both groups) based on our clinical experience. To detect the effect size d of 0.8 between two MAD groups with a power of 80% and a significance level of 5%, a sample size of about 25 patients per group is needed. Therefore, we planned to randomize 25 patients per group.

### Statistical analysis

Normality of continuous data was tested by Shapiro–Wilk test. Independent *t* test (for normally distributed variables), Mann–Whitney *U* test (for non-normally distributed variables), and Chi-squared test or Fisher’s exact test (for nominal variables) were used to compare the baseline characteristics, MAD titration outcomes, MAD response, MAD side-effect reports, and MAD compliance between the MAD-H group and the MAD-S group.

The intra-rater reliability for the upper airway variables was assessed using a two-way mixed, absolute agreement, single measures intraclass correlation coefficient (ICC). Reliability was defined as poor (ICC < 0.5), moderate (ICC = 0.5–0.75), good (ICC = 0.75–0.9), or excellent (ICC > 0.9) [38].

Two-way analyses of variance (ANOVA) were used to compare the mean differences of all outcome variables separately for within-subjects factor (i.e., baseline without MAD versus follow-up with MAD in situ), for between-subjects factor (i.e., the mean of the outcome variables of MAD-H versus MAD-S), and to assess the interaction effect between the two factors (i.e., treatment effect between baseline and follow-up of MAD-H versus MAD-S) on the outcome variables. Any significantly different baseline characteristics between both MAD groups were controlled as covariates in the assessment of the interaction effect [39]. Bonferroni-Holm method was used for the secondary outcome variables to correct for the increased risk of Type I error due to multiple statistical comparisons [40].

A per-protocol (PP) analysis included patients who completed the entire treatment and was performed for all primary and secondary outcomes variables. An intention-to-treat (ITT) analysis included all patients who underwent randomization and was performed only for the primary outcome variables. In ITT analysis, the missing data were imputed by a multiple imputation procedure (five imputed datasets) in SPSS software (SPSS version 20, Chicago, IL, USA). A post hoc power analysis was conducted for the primary outcome variables using the software G*power (version 3.1.9, Franz Faul, Universität Kiel, Germany).

## Results

### Flowchart of participants

**Fig. 4:**
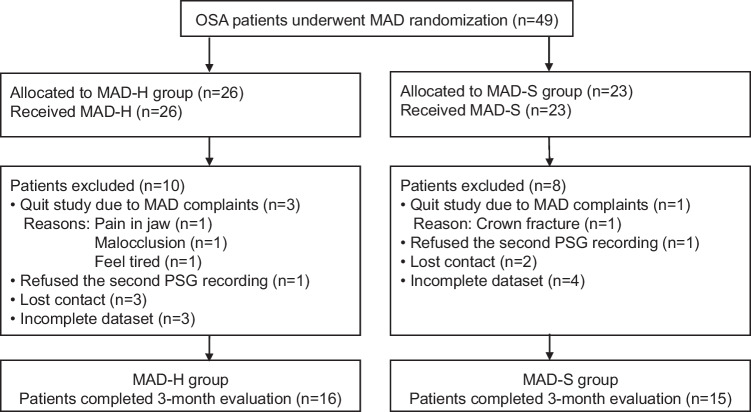
Flowchart of the patients in the study. OSA: obstructive sleep apnea; CBCT: cone beam computed tomography; PSG: Polysomnography; n: sample size; MAD: mandibular advancement device; MAD-H: Herbst appliance; MAD-S: SomnoDent appliance

The flow-chart of 49 patients with OSA who were recruited for this study is shown in Fig. 4. In total, 49 patients underwent MAD randomization and were included in the ITT analysis: 26 patients in MAD-H group and 23 patients in MAD-S group. Of those patients, 10 patients in MAD-H group and 8 patients in MAD-S group were considered as dropouts for the reasons, such as lost contact, quit study, refused the second PSG recordings, and had incomplete dataset. Finally, 16 patients in MAD-H group and 15 patients in MAD-S group completed the entire study and were included in the PP analysis.

### Participant characteristics

The baseline characteristics of the MAD-H group, the MAD-S group, and dropouts are shown in Table 1. There were no significant differences between dropouts and the participants who completed the study in age, gender distribution, body mass index (BMI), neck circumference, and Epworth Sleepiness Scale (ESS) (*P* = 0.30–0.98). The MAD-H group and the MAD-S group did not significantly differ regarding gender distribution, BMI, neck circumference, and ESS (*P* = 0.08–1.00). However, there was a tendency that the MAD-S group (53.3 ± 15.1 years) was significantly older than the MAD-H group (44.0 ± 11.4 years) (*t* =  − 1.94, *P* = 0.06). Therefore, age was controlled as covariate in the later analysis of comparing outcome variables between both MAD groups.

**Table 1:** Baseline characteristics of the MAD-H group, the MAD-S group, and dropouts

	MAD-H (*n* = 16)	MAD-S (*n* = 15)	Dropouts (*n* = 18)	t/FET	*P*
Age (years)	44.0 ± 11.4	53.3 ± 15.1	47.8 ± 12.6	-1.94 (t)	0.06
Gender (men vs women)	11 vs 5	10 vs 5	11 vs 7	FET	1.00
BMI (kg/m^2^)	27.8 ± 4.6	26.5 ± 3.3	25.3|27.2|32.5	0.89 (t)	0.38
Neck circumference (cm)	40.2 ± 3.8	37.7 ± 3.8	39.3 ± 3.3	1.83 (t)	0.08
Epworth Sleepiness Scale ^a^	8.6 ± 3.7	9.1 ± 3.9	8.9 ± 5.1	-0.35 (t)	0.73

Normally distributed data are shown as means ± standard deviations (SD); non-normally distributed data are presented as 25^th^ percentile| median|75^th^ percentile; *t* independent *t* test; *FET* Fisher’s exact test; ^a^: two patients of MAD-H group, one patient of MAD-S group, and 5 patients of dropouts with incomplete ESS score were excluded from the analysis

*MAD-H* Herbst appliance; *MAD-S* SomnoDent appliance; *BMI* body mass index

### MAD use

The titration results, treatment response, side-effects, and compliance of the MAD-H and MAD-S groups are shown in Table 2. No significant difference was found in the mandibular protrusion and mandibular vertical opening after titration between both groups (*P* = 0.54–0.90). Besides, no significant difference was found in the treatment response between both groups (χ^2^ = 0.03, *P* = 0.85). There was no significant difference between both MAD groups in the number of each side-effect (*P* = 0.33–1.00). Seventeen patients responded to the compliance telephone survey, and a few patients counted the wearing hours of MAD prior to their sleep, resulting in wearing hours of more than 100% of their sleep. There was no significant difference between both groups in MAD compliance and satisfaction (*P* = 0.08–0.40).

**Table 2:** The titration results, treatment response, side-effects, and compliance of the MAD-H and MAD-S groups

	MAD-H (*n* = 16)	MAD-S (*n* = 15)	t/Z*/χ*^*2*^*/*FET	*P*
*Titration results*				
Protrusion percentage (%)	75.0|75.0|75.0	60.0|75.0|90.0	-0.62 (Z)	0.54
Protrusion amount (mm)	8.4 ± 2.1	8.7 ± 2.3	-0.43 (t)	0.67
Vertical opening (mm)	11.3 ± 1.5	11.4 ± 1.6	-0.13 (t)	0.90
*Treatment response*				
Responders vs non-responders	8 vs 8	7 vs 8	0.03 (χ^2^)	0.85
*Side-effects*				
Sensitive teeth in the morning	4	1	FET	0.33
Painful jaw muscles	3	1	FET	0.60
Painful temporomandibular complaints	3	3	FET	1.00
Changed occlusion in the morning	3	2	FET	1.00
*MAD compliance*	(n = 10)^a^	(n = 7)^a^		
Wearing hour (%)	100.0|107.1|127.1	100.0|100.0|100.0	-1.75 (Z)	0.08
Wearing day (%)	100.0|100.0|100.0	100.0|100.0|100.0	-0.84 (Z)	0.40
Level of satisfaction (%)	68.3 ± 24.0	77.9 ± 15.8	-0.92 (t)	0.37

Normally distributed data are shown as means ± standard deviations (SD); non-normally distributed data are presented as 25^th^ percentile| median|75^th^ percentile; ^a^:10 patients in MAD-H group and 7 patients in MAD-S group responded to compliance telephone survey; *t* independent t test; *Z* Mann–Whitney *U* test; χ^2^: Chi-squared test; *FET* Fisher’s exact test

*MAD-H* Herbst appliance; *MAD-S* SomnoDent appliance; *Wearing hour* percentage of hours of MAD use per total sleep time, and a few patients counted the wearing hours of MAD prior to their sleep, resulting in wearing hours of more than 100% of their sleep; *Wearing day* percentage of days of MAD use per week; *Level of satisfaction* based on a visual analogue scale of 0 to 100, where 0 was unsatisfied, and 100 was very satisfied

### Sleep and respiration

**Fig. 5:**
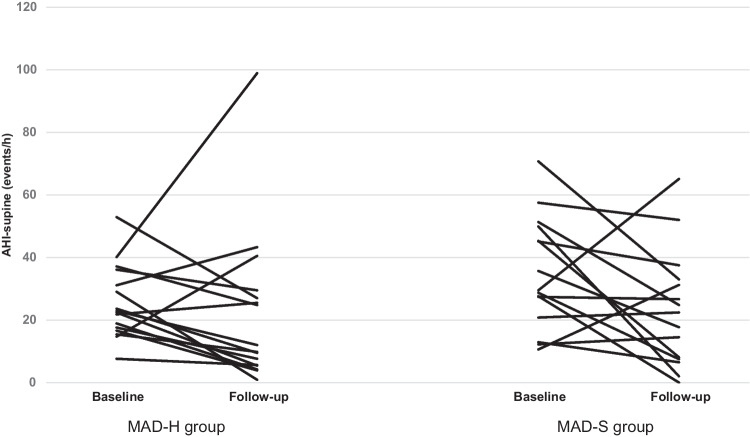
The individual effects of MAD-H (*n* = 16) and MAD-S (*n* = 15) on AHI-supine. MAD: mandibular advancement device; MAD-H: Herbst appliance; MAD-S: SomnoDent appliance; AHI-supine: apnea–hypopnea index in supine position

The sleep and respiratory variables without and with MAD in situ of the MAD-H group and the MAD-S group are presented in Table 3. For the primary outcome variable AHI-supine, both PP and ITT analyses indicated that there was no significant difference between MAD-H and MAD-S in improving the AHI-supine (*P* = 0.14 and *P* = 0.47, respectively). The individual effects of both MADs on AHI-supine are illustrated in Fig. 5. According to the post hoc power analysis, the effect size f of the AHI-supine was 0.29 in the PP analysis and was 0.10 in the ITT analysis (partial η^2^ = 0.08 and 0.01, respectively), which is qualified as small to medium. Besides, both PP analysis and ITT analysis indicated that there was no significant change in AHI-supine with MAD in situ in the total group (*P* = 0.06 and* P* = 0.12, respectively). For the secondary variables, there was no significant difference between the effects of MAD-H and MAD-S (all *P* > 0.05). However, the AHI, AHI-non-supine and ODI reduced significantly with MAD in situ in the total group (*P* < 0.01, *P* = 0.01, and *P* = 0.02, respectively).

**Table 3:** The sleep and respiratory variables without and with MAD in situ of the MAD-H group and the MAD-S group

	MAD-H group (*n* = 16)		MAD-S group (*n* = 15)		Baseline MAD-H vs MAD-S	Within-subjects effect (Baseline vs follow-up in the total group)		Interaction effect ^a^ (MAD-H vs MAD-S in treatment effect)	
	Baseline	follow-up	Baseline	follow-up	*P*	F	*P*	F	*P*
*Primary outcome*									
AHI-supine (/h)	25.5 ± 11.6	21.8 ± 24.6	35.0 ± 17.9	23.3 ± 18.5	0.09	3.85	0.06	2.26	0.14
*Secondary outcomes*									
Sleep variables									
Total sleep time (min)	420.5 ± 56.6	415.6 ± 67.1	378.0 ± 63.7	388.5 ± 75.9	0.06	0.04	0.85	0.16	0.69
Stage N1 (%)^b^	9.3 ± 5.4	9.1 ± 6.6	8.2 ± 4.8	4.8 ± 3.3	0.56	2.37	0.14	2.92	0.10
Stage N2 (%)^b^	50.9 ± 7.8	48.9 ± 11.1	53.3 ± 9.6	46.9 ± 12.3	0.97	2.67	0.11	0.55	0.47
Stage N3 (%)^b^	18.6 ± 6.7	19.6 ± 7.3	17.9 ± 6.8	23.9 ± 9.8	0.79	4.14	0.05	2.10	0.16
Stage REM (%)^b^	21.1 ± 5.7	22.5 ± 6.6	20.6 ± 4.7	24.4 ± 6.8	0.78	2.33	0.14	0.65	0.43
Supine sleep time (%)	53.6 ± 33.2	48.9 ± 26.6	33.5 ± 29.8	30.0 ± 26.1	0.06	0.60	0.45	0.88	0.36
Arousal index (/h)^b^	7.6 ± 6.9	14.2 ± 14.0	12.5 ± 15.7	11.1 ± 10.6	0.50	0.62	0.44	1.30	0.27
Respiratory variables									
AHI (/h)	16.2 ± 6.1	11.4 ± 7.7	17.1 ± 7.5	11.1 ± 8.7	0.69	12.55	< 0.01*	1.82	0.19
AHI-non-supine (/h)	7.1 ± 7.1	5.5 ± 4.4	12.4 ± 8.9	4.8 ± 3.3	0.07	8.49	0.01*	0.85	0.37
ODI (/h)	16.5 ± 9.7	11.0 ± 6.9	15.6 ± 10.7	12.3 ± 7.4	0.91	6.13	0.02*	0.00	0.95

Data are shown as means ± standard deviations (SD); ^a^: all outcome variables were controlled for age, and the variables of total sleep time, supine sleep time, and AHI-non-supine were controlled additionally for their baseline values; ^b^:two patients in MAD-S group with incomplete sleep dataset were excluded from the analysis of sleep variables; *: significant difference after Bonferroni-Holm correction

*AHI* apnea–hypopnea index; *AHI-supine* AHI in supine position; *AHI-non-supine* AHI in positions other than supine position; ODI: oxygen desaturation index; REM: rapid eye movement

### Reliability of the upper airway assessment

The intra-rater reliability for the upper airway assessment was excellent, with ICC = 0.96 for the CSAmin and ICC = 0.90 to 0.94 for the secondary outcome variables.

### Upper airway dimensions

The upper airway dimensions without and with MAD in situ of the MAD-H group and the MAD-S group are presented in Table 4. For the primary outcome variable CSAmin, both PP analysis and ITT analysis showed similar results: there was no significant difference between MAD-H and MAD-S in increasing the CSAmin (*P* = 0.59 and* P* = 0.57, respectively). According to the post hoc power analysis, the effect size f of the CSAmin was 0.10 in the PP analysis and was 0.08 in the ITT analysis (partial η^2^ = 0.01 and 0.007, respectively), which is qualified as small. Besides, both PP analysis and ITT analysis indicated that the CSAmin increased significantly with MAD in situ in the total group (*P* = 0.01 and* P* < 0.01, respectively). For the secondary outcome variables, there was no significant difference between the effects of MAD-H and MAD-S (all *P* > 0.05). However, the lateral dimension of the CSAmin (La) increased significantly with MAD in situ in the total group (*P* < 0.01).

**Table 4:** Upper airway dimensions without and with MAD in situ of the MAD-H group and the MAD-S group

	MAD-H group (*n* = 16)		MAD-S group (*n* = 15)		Baseline MAD-H vs MAD-S	Within-subjects effect (Baseline vs follow-up in the total group)		Interaction effect ^a^ (MAD-H vs MAD-S in treatment effect)	
	Baseline	follow-up	Baseline	follow-up	*P*	F	*P*	F	*P*
*Primary outcome*									
CSAmin (mm^2^)	54.8 ± 36.9	75.6 ± 50.4	58.5 ± 29.3	87.8 ± 59.1	0.50	9.13	0.01*	0.30	0.59
*Secondary outcomes*									
A-P (mm)	4.6 ± 2.1	5.0 ± 2.4	4.4 ± 1.7	5.3 ± 1.9	0.77	3.37	0.08	0.35	0.56
La (mm)	11.4 ± 4.7	15.4 ± 5.1	13.1 ± 4.6	16.2 ± 5.8	0.32	16.74	< 0.01*	0.12	0.74
L (mm)	67.0 ± 9.6	65.6 ± 9.9	65.9 ± 8.0	64.4 ± 8.8	0.59	4.30	0.05	0.00	0.99
V (cm^3^)	10.9 ± 3.4	12.2 ± 5.5	11.4 ± 5.4	12.8 ± 5.7	0.75	3.42	0.08	0.10	0.75

Data are shown as means ± standard deviations (SD); ^a^: all outcome variables were controlled for age; *: significant difference after Bonferroni-Holm correction

*CSAmin* minimum cross-sectional area of the upper airway; *A-P* anterior–posterior dimension of the CSAmin; *La* lateral dimension of the CSAmin; *L* length of the upper airway; *V* upper airway volume

## Discussion

The aim of this RCT was to compare the effects of MAD-H (allowing limited vertical opening) and MAD-S (allowing free vertical opening) on respiratory parameters and upper airway dimensions in patients with mild to moderate OSA. The results showed that despite differences in the freedom of mandibular vertical opening, there was no significant difference between MAD-H and MAD-S in improving the respiratory parameters and upper airway dimensions.

### MAD-H vs MAD-S

In the present study, there was no significant difference between the MAD-H and MAD-S in affecting the AHI-supine, as well as AHI and AHI-non-supine. According to the post hoc power analysis, the effect size f of the AHI-supine was 0.29 in the PP analysis and was 0.10 in the ITT analysis, which is qualified as small to medium (an effect size f = 0.10 is small, one = 0.25 is medium, and one = 0.40 is large [37]). With this effect size, an enlargement of our total sample size to around 100–800 patients, is needed to find a statistically significant difference between both groups (with power 0.8; 5% significance level), which makes the clinical relevance of such a finding questionable. Regarding the clinical relevance of two interventions, the concept of the number needed to treat (NNT) is often used. In this study, 50% in MAD-H group and 47% in MAD-S group were treated successfully. The NNT is 33, implying that about 33 patients with mild to moderate OSA need to be treated with MAD-H to get one more successfully treated patient as compared to treatment with MAD-S, which is not clinically relevant [41]. Although the freedom of vertical opening is different between MAD-H and MAD-S, it seems that the respiratory outcomes were not affected by this design feature. The same protrusion and vertical opening of the mandible in awake state may explain the similarity in treatment efficiency between MAD-H and MAD-S. However, as we did not measure the vertical opening in sleep, future studies are warranted to verify this hypothesis. Similar to our study, a retrospective study of Verburg et al. [21] also compared MAD-H and MAD-S and found no significant difference in improving the AHI. Thus, an MAD allowing limited vertical opening and an MAD allowing free vertical opening may result in similar respiratory outcomes.

No significant difference was found between both MADs in changing the upper airway dimensions. With the same protrusion position and vertical opening, the same effect of MAD-H and MAD-S was applied to the tongue and mandible in the awake state, and thus no difference of upper airway changes could be found between both groups. Our results are similar to the outcomes of two previous studies based on cephalometric images [16, 42], in which no significantly different effects on the upper airway dimensions were found when two MADs with similar protrusion and vertical opening were compared. However, limited to 2-D radiology images, those studies only compared the upper airway in anterior–posterior direction [16, 42]. By using CBCT images, our study has the advantage of providing additional information on lateral direction dimension, CSAmin, and volume of the upper airway. The current CBCT scanning and measurement protocols were standardized, and have been proven to have an excellent reliability in the upper airway assessment in the present study (ICC = 0.90 to 0.96) as well as in our previous study [36]. However, a study of Ryan et al. [43] has suggested that different CBCT scanning timings of the same patient with same scanning and patient positioning protocols can result in different upper airway volumetric data, suggesting the intra-individual variability in CBCT assessments. However, this intra-individual variability was randomly present in both groups, and therefore may not explain our non-significant findings in the comparison between groups.

The self-reported side effects were not significantly different between both MAD groups. However, sensitive teeth and painful jaw muscles were 3–4 times more frequent in the MAD-H group compared to MAD-S group, which might be due to the different design feature. However, it seems that the side effects of MAD in most cases were of a minor nature and probably improve in the longer term [44, 45]. A study with a long-term is needed to confirm the clinical relevance of our short-term finding.

### Overall MAD effects

For respiratory parameters, there was significant decrease in the AHI and AHI-non-supine but no significant decrease in the AHI-supine with MAD in situ in the total group, which indicated that the MADs used in this study were less effective in reducing the AHI in supine position. As MADs used in this study allow more or less freedom of vertical opening, gravity would favor mouth opening and weaken the beneficial effects of mandibular protrusion in supine position for both MADs. A study of Milano et al. [46] compared a group using an MAD allowing free vertical opening and a group using the same MAD with elastics to restrict the vertical opening, and suggested that the AHI-supine was improved significantly in both groups but that the improvement of AHI-supine was significantly greater in the group MAD with elastics. However, the patients in their study were positional OSA, which represents a different study sample as compared to our study and may explain the different effects of MAD allowing free vertical opening on AHI-supine. Inserting an MAD did not significantly change the time spent in supine position and REM stage, which is consistent with other studies [18, 47]. Given that sleeping in supine position and REM stage may worsen the respiratory events [48], the treatment effects of MAD in the present study were not related to the time spent in supine position and/or REM stage at therapy evaluation.

The treatment response rate in the present study (48%) is in line with other studies reporting MAD response rate between 43 and 77% [49]. Using the same titration protocol and response rate criteria as the present study, the study of de Ruiter et al. [31] reported a 44.4% treatment response rate, which is in line with our results as well.

The CSAmin increased significantly with MAD in situ in the total group. Further, the increase of CSAmin was mainly due to the enlargement in the lateral dimension, which is similar to previous studies [50–52]. Although an MAD works primarily by protruding the mandible and tongue, the enlargement of the upper airway was predominantly in the lateral dimensions. The precise mechanism for this observation is not completely understood, but it has been suggested that this is related to the soft tissue connections between the tongue, soft palate, and lateral pharyngeal walls through the palatopharyngeal and palatoglossal arches [51]. Mandibular advancement possibly stretches the lateral pharyngeal walls through these soft tissue connections, and results in lateral enlargement of the CSAmin.

### Limitations

The present study has several limitations. Firstly, the information of the patients who were initially screened for this study was missing. With these data missing, we cannot be certain that our sample represents the total population of OSA patients that are being seen in the clinics in Amsterdam. Although the dropout rate (36%) seemed high in our study, the dropout rate was comparable to our previous studies [33, 53]. Importantly, there were no significant differences between dropouts and the participants in baseline characteristics. Further, the intention-to-treat analyses showed similar results as the per-protocol analyses. Secondly, the upper airway images were taken in awake state, and therefore the upper airway morphology may not be identical to the sleep state. Besides, CBCT assessment while awake do not reflect the actual effects of MAD on the upper airway in sleep as both types of MADs allow more or less mouth opening in sleep. However, it is currently challenging to scan the upper airway during sleep. As to resemble the sleep state as much as possible, patients were instructed to relax their masticatory muscles with the MAD in situ when performing the follow-up CBCT scans. Importantly, since the CBCT scans at baseline and at therapy evaluation were both performed in awake supine condition, the effects of MADs on upper airway dimensions were unbiased. Thirdly, only a part of patients responded to the compliance telephone survey (response rate: 55%). It could be possible that patients who responded to the telephone survey in the present study were more willing to use MAD, resulting an overestimation of MAD compliance. Besides, the intensive follow-up titration protocol may also have contributed to a high compliance. Finally, subjective treatment outcomes, including snoring, daytime sleepiness (Epworth Sleepiness Scale), and quality of life, are also important therapeutic targets in patients with OSA, and therefore future studies should involve these aspects. Besides, as the present study investigated the treatment effect of both MADs in a short-term, a long-term follow-up study is needed to confirm our short-term findings.

### Strengths

The standardized titration protocol used for both types of MAD is one of the major strengths of the present study. To the best of our knowledge, when comparing different MADs, few previous studies have reported a standardized titration protocol. In the present study, a detailed standardized titration protocol was applied, and no significant difference was found in finial protrusion position of both MAD groups, which enables an unbiased comparison. The standardized stepwise titration protocol for MAD, developed by one of the co-authors of this paper (GA), has been proved to have good efficacy, good tolerance, and good adherence [31], and is therefore recommended for use in clinical studies and practices.

Inserting an MAD intra-orally induces a certain amount of mandibular vertical opening. A study of Mayoral et al. [54] has indicated that as the vertical dimension increases, the mandible rotates posteriorly and places itself in a more retrusive location (0.3 mm for every 1 mm of vertical increase). Therefore, it is recommended to limit the vertical opening of MADs [54, 55]. In this study, the vertical opening for MAD-H and for MAD-S in awake state were similar. To guarantee an accurate measurement of the vertical opening, we added the overbite and inter-incisor distance with MAD in situ together. As most previous studies only used inter-incisor distance to estimate the vertical opening, the accurate measurement of the vertical opening is another strength of the present study.

## Conclusions

Within the limitations of this study, we conclude that there is no significant difference between the effects of an MAD allowing limited vertical opening and an MAD allowing free vertical opening on respiratory parameters and upper airway dimensions in patients with mild to moderate OSA.

## Data Availability

The datasets generated and/or analyzed during the current study are available from the corresponding author on reasonable request.
